# Headache after exposure to ‘date-rape’ drugs

**DOI:** 10.1186/2193-1801-2-39

**Published:** 2013-02-08

**Authors:** Richard Peatfield, Carlos M Villalón

**Affiliations:** 1 Princess Margaret Migraine Clinic, Charing Cross Hospital, London, W6 8RF UK; 2 Departamento de Farmacobiología, Cinvestav-Coapa, Deleg. Tlalpan, 14330 México D.F, Mexico

## Abstract

**Summary:**

We report two patients who developed a prolonged featureless headache, they think after a drink was ‘spiked’. We speculate that each was exposed to scopolamine, resulting in enhanced trigeminal release of vasodilator neuropeptides, including Calcitonin Gene-Related Peptide (CGRP), and thus the headache.

## Introduction

Many hypnotic and tranquillising drugs can also induce amnesia, an effect exploited by anaesthetists when performing potentially distressing medical procedures such as endoscopies in anxious patients. Unfortunately, many of the drugs have also found a place in criminal youth culture, enabling the unscrupulous to render victims powerless to resist sexual assault or even theft. As a result some of these agents, including flunitrazepam (Rohypnol) have been withdrawn from sale. Many are benzodiazepines, and are sedative, which is often useful in legitimate clinical practice, but (it may be assumed) less so when such drugs are used for criminal purposes.

The muscarinic receptor antagonist scopolamine (l-hyoscine), derived from Henbane in Europe, has been used as an anaesthetic premedication for many years, as it simultaneously inhibits vagal reflexes, dries oral secretions and impairs memory, without suppressing respiration. Unfortunately, it can also induce restlessness, excitement, hallucinations and euphoria. Indeed, its affinity for subtypes of the muscarinic acetylcholine receptor seems little different from the closely related drug atropine, but due to its higher lipid solubility it penetrates the central nervous system much better (Bonner et al. 1987). It is found in larger quantities in a number of South American plants, most notably the Borrachera tree, yielding Burundanga “the terror of Colombia”. When taken orally, Burundanga (pronounced boor-oon-DAN-ga) induces a hypnosis-like state, and it is becoming the assault-chemical of choice there, due to its disinhibitory and amnesic effects.

In the present study, we report two young women who each behaved in an uncharacteristic manner after an evening out in London, and who then developed a chronic, relatively featureless headache, and speculate that this might have been a result of exposure to scopolamine.

## Case 1

A 24 year old woman, who works as a travel manager for a theatre management company; said that she often goes to social occasions as part of her work. She had had migraines since her menarche at the age of 12. The attacks were first infrequent, but recently they had been occurring every six to seven weeks, lasting a day. It was a bifrontal throbbing headache with a little nausea (but no vomiting), and with photophobia but no other focal symptoms. The pain usually responded to Zolmitriptan. Her mother, maternal grandmother, aunt and older brother were similarly affected.

She developed a chronic headache in September 2009. This had started the night after a work dinner, at which she emphasises that she only had one alcoholic drink. Towards the end of the evening, she suddenly started slurring her speech, and a senior colleague persuaded her to go home. She has absolutely no memory of her journey back to (she presumes) Charing Cross station, perhaps a 5 minute walk. She seems to have brought a train ticket as judged by her own credit card account (even though she holds a season ticket to Dartford), and found herself in Dover at 3 am with no intervening memory. She got a taxi home, but then had to be looked after by a friend as her parents were abroad. She was quite unable to move for a full day. It is of great interest that several of her colleagues (who had never hitherto been interested in her welfare) phoned her at home to ensure that she had got home safely.

She had a generalised headache from then, like a “squeezing pain” right through her head. This failed to respond first to Zolmitriptan, and then to Co-codamol, and more recently she has taken amitriptyline (25 mg daily), which has improved her sleeping pattern, but not the headache, though it does vary in severity. She discontinued the contraceptive pill (Microgynon) in late August 2009 because of weight gain. She still sleeps poorly, with insomnia for up to two to three hours, but no early waking. There were no significant abnormalities on examination. The dose of amitriptyline was increased and the codeine discontinued, depending as best she can on anti-inflammatory drugs.

## Case 2

This 18 year old woman works as a sales assistant in a jewellery shop. There was no past or family history of headache. She thinks a drink was spiked “by the barman” in a night club in March 2009. Her mother's account is that she was “put outside” the night club where she collapsed, and her mother came to fetch her. She was found to be drowsy and crying with her eyes wide open. She could not see her mother, but recognised her voice. She was taken to the local hospital emergency department, but treated as a “drunken teenager” and allowed to sleep it off in a cubicle. She was clearly difficult to rouse during this time. She was completely amnesic until waking up in hospital, perhaps five hours after she took the drink.

For the next eighteen months she had a chronic, though variable, right hemicranial headache. There was some nausea, and occasional vomiting when the pain was bad. She was not pregnant. She had taken Microgynon 30 since the age of 13, and she had used dothiepin at a dose of 75 mg daily for the headache without much benefit. The pain had not responded to paracetamol, co-codamol or tramadol.

There was a slight drift of the right hand on examination, but no other abnormalities. An MRI scan showed no abnormality in the brain substance, though there was a glomus vagale paraganglioma, which was considered asymptomatic, and is being monitored with regular scans. A trial of indomethacin was unsuccessful, as was topiramate up to 100 mg daily. She discontinued this in September 2010, and the pain has gradually improved since then. By June 2011 she was having a 4-day attack every 2–3 months.

## Discussion

There seems to be at least circumstantial evidence these women's drinks were 'spiked', though identification of the drug administered is necessarily speculative. We have no direct evidence at all, though the relative lack of sedation does suggest Burundanga.

Burundanga is a kind of voodoo powder obtained from a Colombian local plant of the nightshade family, a shrub called borrachera, or “drunken binge”. Used for hundreds of years by native South Americans in religious ceremonies, the powder when ingested causes victims to lose their will and memory, sometimes for days. When refined, the powder yields scopolamine, a well-known drug with legitimate uses as a sedative and to combat motion sickness. But in Colombia the drug’s most avid users are street criminals. Crooks mix the powder with sedatives and feed the Burundanga cocktail to unsuspecting victims whom they then proceed to rob - or worse. Doctors there estimate that Colombian hustlers slip the odourless, colourless and soluble Burundanga in food or drink to about 500 unwitting victims in the city each month. About half of the city’s total emergency room admissions for poison are Burundanga victims (De Cordoba 1995).

Cases of headache induced by scopolamine have been recorded. Gordon (Gordon et al. 1991) reported a naval airman who developed chronic headaches after five months’ continuous treatment with scopolamine, and Ikeda et al. (Ikeda et al. 2009) noted that 54 out of 1919 subjects premedicated with 20 mg scopolamine for‚ gastrointestinal X-ray examination’ developed headaches - all had a past history of migraine, and only one of the 1865 non-migraineurs had a headache, and this was mild. The headaches lasted several hours. The headache seems unrelated to the suppression of muscarinic parasympathetic vasodilatation in the cerebral circulation, as scopolamine does not affect this, at least in the rat (Kowacs et al. 2004). The therapeutic benefit of acetylcholinesterase inhibitor drugs such as donepezil in migraine (Ikeda et al. 2009; Nicolodi et al. 2002) suggests that cholinergic pathways may suppress migrainous attacks and, thus, muscarinic cholinergic receptor antagonists might lead to the development of headache.

Animal studies suggest that M_2_ muscarinic receptor subtypes are presynaptically located on cholinergic nerve terminals, where they can act as autoreceptors to decrease acetylcholine release (by negative feed-back mechanisms). Interestingly, blockade of these sites (with atropine, scopolamine or more selective M_2_ receptor antagonists) facilitates acetylcholine release and learning in rats (Vazquez & Baghdoyan 2003). Although we did not directly measure CGRP concentrations in the jugular venous blood of these women, we wonder if prejunctional muscarinic blockade (by scopolamine) may have led to an increase in CGRP release. This would follow from the fact that muscarinic M2 and M4 receptors (for which scopolamine displays a high affinity) are coupled to Gi proteins; upon activation, this would lead to a decrease in intraneuronal cAMP levels, a decrease in Ca2+ conductance and an increase in K + conductance. These are signal transduction systems usually associated with inhibition of neurotransmitters release.

If this were the case, then blockade of these receptors by scopolamine could result in an increase in CGRP release, cranial vasodilatation and, consequently, headache, or even migraine! The actions of scopolamine are longer lasting than those of atropine, though clearly some long-lasting adaptive mechanism is required to account for a headache that lasts for several months.
